# The role of emotions in depression and aggression

**DOI:** 10.4317/medoral.21561

**Published:** 2016-07-31

**Authors:** Anna Llorca, Elisabeth Malonda, Paula Samper

**Affiliations:** 1 Departmento de Personalidad, Evaluación y Tratamientos psicológicos, Universitat de València, Avda. Blasco Ibáñez, 21, 46010 Valencia, Spain; 2 Departmento de Psicología Básica. Universitat de València

## Abstract

**Background:**

Depression is a broad and heterogeneous diagnostic grouping, central to which is depressed mood or inability to enjoy most activities. Depressive symptoms are frequently accompanied by conduct problems stemming from anger. It is very important to know the interrelation of these emotions very well to be able to help adolescents to manage them more easily. The main aim of this article is to present the problem of interaction between negative affects (emotional instability, anger state and trait, physical and verbal aggression and depression) analyzing the different relationship through the time in spanish sample.

**Material and Methods:**

The sample included 470 adolescents (192 boys and 225 girls) in a three-wave longitudinal study in Valencia (Spain). The mean age was 14.70 in the first wave. Structural equations modelling was employed to explore two longitudinal models.

**Results:**

The results show differences based on sex, and that an internalised variable, like emotional instability, is relevant to prevent the appearance of depression directly in girls and also the later appearance of aggression as long as anger mediates, in both boys and girls, so the control of anger becomes an important goal to control the rest of the negative affects.

**Conclusions:**

This results has consequences in the preparation of all programmes that try to establish an emotional control on adolescents, as not only has to be taken into account as a direct goal the control of externalised emotions like anger, but internalised emotions like emotional instability have to be taken into account also. Furthermore, it is also made apparent that not only the punctual explosions and externalisation of anger have to be worked on, but the temperamental aspects which are the base of anger trait have to be worked on too.

**Key words:**Emotional instability, anger, depression, aggression, negative emotions, adolescence.

## Introduction

Children and teenagers can experience emotional disorders like depression, anger or anxiety. Sometimes it can be difficult for adults to understand children’s problems because we look at them through adult eyes and we don’t understand what is wrong in their lives, but it is important to take problems in young people seriously, because many studies underline their permanence (1).

Depression is a broad and heterogeneous diagnostic grouping, central to which is depressed mood or inability to enjoy most activities. Depressive symptoms are frequently accompanied by symptoms of anxiety, and conduct problems stemming from anger.

Many people who experience together depression, anxiety, feel irritable or angry, especially teenagers, normally they can’t interpret nor express their emotional changes appropriately. It is very important to know the interrelation of these emotions very well to be able to help adolescents to manage them more easily. The relationship among these variables is essential to achieve this goal, but the results obtained in several studies about this subject differ from one another due to the use of different methodologies and sources or due to the characteristics of the population being studied (2,3), all of the above difficults the reliability of the results.

Another element to be clarified is the role of gender in this relationship between emotions during adolescence as the results aren’t consistent; some authors maintain that there are differences while others don’t believe there are any (4).

One of the added problems is the comorbidity very frequent in children and adolescents. About half the people diagnosed with depression are also diagnosed with an anxiety disorder (5,1) and the comorbidity between anger and depression is even higher and it presents enough of a problem in research (6). The comorbidity between depression and conduct problems is also very high (7).

There are many different reasons that explain these emotional factor disorders, some of them are intrinsic to the child, like personality (8), and others are related to the context, especially to the family (9) or parenting (10). All these risk factors are important, but it is specially important the relationship between externalised and internalised conducts throughout time (11) as it allows preventive intervention. In relation to this problem, many authors defend that externalised problems predict internalised problems (12), but, in some cases a reverse prediction appears, for example, depression predicts aggression in adolescents (13) and it is precisely this that is going to be explored here, the relationship between these constructs. Therefore, the aim is not the search for causes but the analysis of the dynamics of interaction between these emotional variables to be able to contribute which one has a greater explanatory power in relation to the rest and its longitudinal permanency as many studies suggest that prediction through time is not possible especially with adolescent depression (11).

The aim of this article is plural. First we present the problem of interaction between negative affects (emotional instability, anger state and trait, physical and verbal aggression and depression) analyzing the different relationship through the time in our sample. The second goal will be to contribute to the managing of the negative affections depending on their relation to other elements (14) and this way to be able to delineate the most effective protection when facing this type of alterations (15).

## Material and Methods

- Participants

Four hundred and seventeen adolescents participated in a three-wave longitudinal study in Valencia, Spain. The sample consisted of 192 boys and 225 girls. In the first wave, adolescents were either in the third year of secondary school (81 boys and 85 girls) or the fourth year of secondary school (111 boys and 140 girls). The mean age was 14.70 (SD = 0.68; range = 13-17 years). This study monitored participating adolescents for three years.

Most adolescents came from two-parent households where parents were married (83.7% married; 13.2% divorced). In terms of educational attainment, 21.8% of mothers had less than a secondary school diploma, 42.2% had a secondary school diploma or equivalent and 30.7% had some university education. Similarly, 24% of fathers had less than a high school diploma, 41% had a high school diploma or equivalent and 28.7% had some university education. Most students self-identified themselves as being from Spain (86.6%). Small percentages of the remaining students self-identified themselves as being from Latin America (e.g., 3.4% from Ecuador, 2% from Colombia and 1.1% from Bolivia) and Eastern European countries (e.g., 1.7% from Romania). Participating schools were randomly selected from the list of all schools in Valencia with students enrolled in compulsory secondary education. In total, 11 schools participated in the study.

- Procedure

Approval from the School Council and parental consent were obtained. Participation by students was voluntary; students were free to decline to participate. The survey was administered by trained researchers in the classroom in 50-minute sessions during school hours. The annual assessments took place in three successive years during the first trimester of the school year. The study followed all ethical guidelines, respecting respondents’ anonymity for both data collection and data analysis.

- Measures

Physical and Verbal Aggression Scale (AFV) (16,17). This instrument uses 20 items to evaluate behaviours that harm others physically or verbally. Cronbach’s alpha for this study was .80 at wave 1, .82 at wave 2 and .83 at wave 3).

Emotional Instability Scale (EI) (16,17). It describes the behaviour that indicates a lack of self-control in social situations as a result of the scarce ability to curb impulsiveness and emotionality). Cronbach’s alpha for this study was .82 at wave 1, .79 at wave 2 and .83 at wave 3).

State and Trait Anger Scale (STAXI-N) (18). It evaluates the anger as a state (α = .76 at wave 1; α = .88 at wave 2; α = .90 at wave 3) and trait (α = .74 at wave 1; α = .76 at wave 2; α = .77 at wave 3).

CES-D Scale (19). This scale assesses levels of depressive symptomatology. Cronbach’s alpha for this study was α = .75 at wave 1; α = .82 at wave 2; α = .82 at wave 3).

- Statistical procedure

First, SPSS 19 was used to calculate means and standard deviations and to perform repeated measures analysis of variance (ANOVA) to test for mean differences across waves and genders. Correlation analysis was carried out to test the relationships among variables. Finally, structural equations modelling (SEM) in AMOS 17.0 (SPSS Inc., 2007) was employed to explore two longitudinal models. The following goodness-of-fit indexes were used: chi-square, chi-square divided by degrees of freedom (c2/d.f.), goodness-of-fit index (GFI), adjusted goodness-of-fit index (AGFI) and Bentler comparative fit index (CFI). Root mean residual (RMR) and root mean square error of approximation (RMSEA) were used to measure error.

## Results

- Repeated Measures Analysis

 Table 1 presents the measurements and typical deviation of the physical and verbal aggression, depression, anger state and trait and emotional instability throughout the time and by sexes.

**Table 1:**
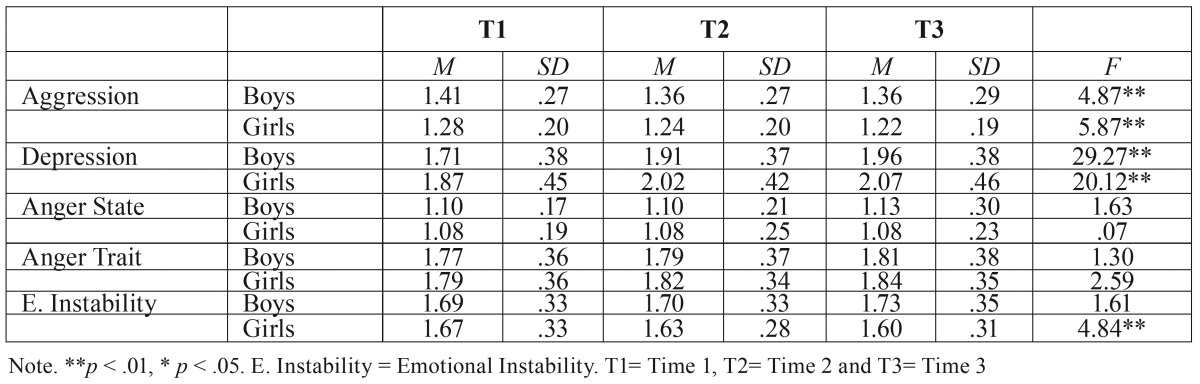
Means and Standard Deviations throughout the time and by sexes.

If we take into consideration how the gender factor works in these relations we can observe ( Table 1) that physical and verbal aggression lowers through the time significantly in boys and girls. In the boys the differences are considerably between T1 and T2 and in the girls between T1 and T2 and between T1 and T3. Having said that, the boys score higher in the three intervals (T1: F = 29.88; *p* < .001; T2: F =29.51; *p* < .001; T3: F = 31.72; *p* < .001).

Regarding depression, it increases significantly in time in both boys and girls. Also, the differences are significant between T1 and T2 and between T1 and T3, in both sexes. Contrary to what happened with aggression, the girls score higher in the three intervals (T1: F = 14.65; *p* < .001; T2: F = 7.29; *p* < .05; T3: F = 5.91; *p* < .05).

Regarding emotional instability, while in the boys it rises slightly in time (not significantly), in the girls it lowers significantly in interval 2 and 3. In the girls, the differences are significant between T1 and intervals 2 and 3. In T2 (F = 5.13; *p* < .05) y 3 (F = 15.23; *p* < .001) the boys score higher than the girls in emotional instability. Likewise, there aren’t significant differences in T1 (F = .40; *p* > .05).

Finally, there aren’t any significant differences in anger state, anger trait throughout the time and depending on gender in any of the evaluated intervals (Anger state, T1: F = 1.80; *p* > .05; T2: F = .35; *p* > .05; T3: F = 3.14; *p* > .05; Anger trait, T1: F = .22; *p* > .05; T2: F = .88; *p* > .05; T3: F = .69; *p* > .05). However, in both sexes, anger tends to increase slightly.

- Correlations

The results show first of all (see  Table 2), that depression in interval 3 correlates with aggression in the three intervals in boys, while in girls, depression in interval 3 relates to aggression in T2 and T3. Also, depression in T3 relates, in boys, with emotional instability from the first and second intervals, and in girls, with emotional instability in the second and third interval.

**Table 2:**
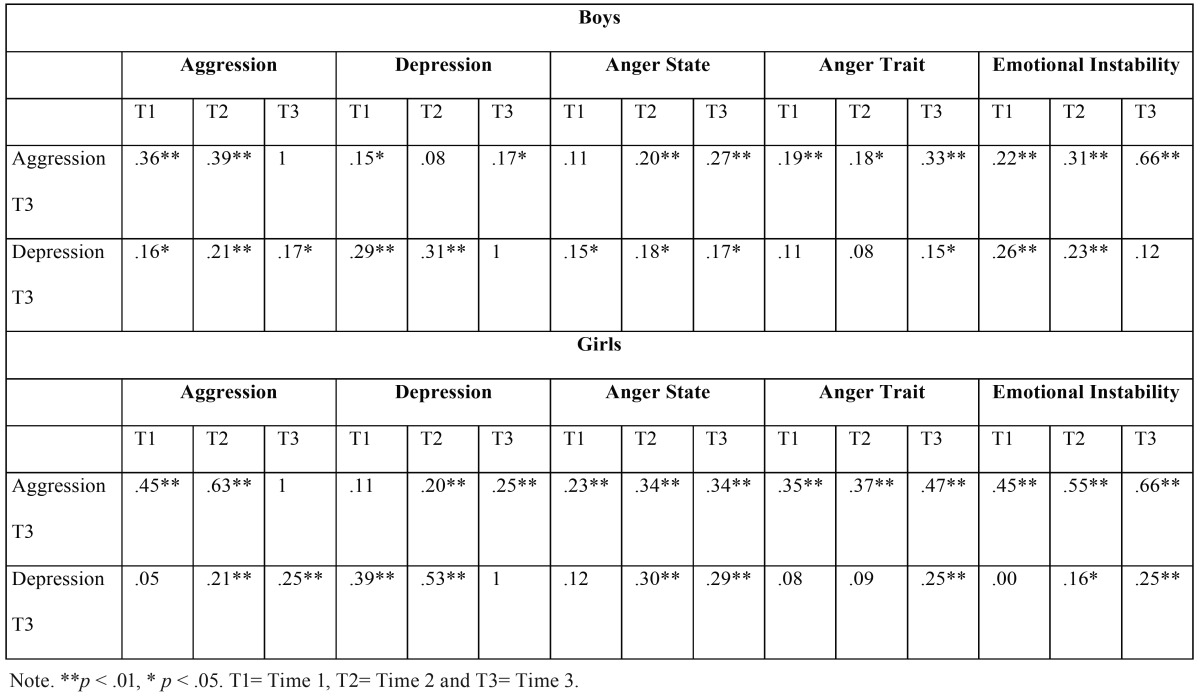
Correlations by sexes.

On the other hand, physical and verbal aggression in interval 3 relates to depression in T1 and T3 in boys and in T2 and T3 in girls. Furthermore, aggression in the third interval relates to anger state and anger trait in all intervals, both in boys and girls. These relations are higher in girls than boys. Finally, aggression in T3 relates to emotional instability in the three intervals in both sexes, although to a greater extent in girls.

The more powerful correlations appear between physical and verbal aggression and emotional instability in any of the three intervals evaluated. Likewise, physical and verbal aggression is strongly related to anger trait and state. In all cases, the correlations are more powerful the closer they are in time. It is interesting to point out that the correlation between depression and anger reaches important levels, above all in the case of anger state in girls.

- Test of longitudinal model

Differences exist between both boys and girls in all variables except for anger trait. Therefore, we consider more useful the study of structural dynamics separating boys and girls.

Two longitudinal path analyses were tested using AMOS 17 and maximum likelihood estimation with robust standard errors, one for boys and one for girls. The model test yielded acceptable fit to the data, c2 (6) = 14.73, *p* <. 05, c2 /fd = 2.45; GFI= .98; AGFI = .93; CFI = .96; RMSEA = .059 and RMR = .005.

**Figure 1:**
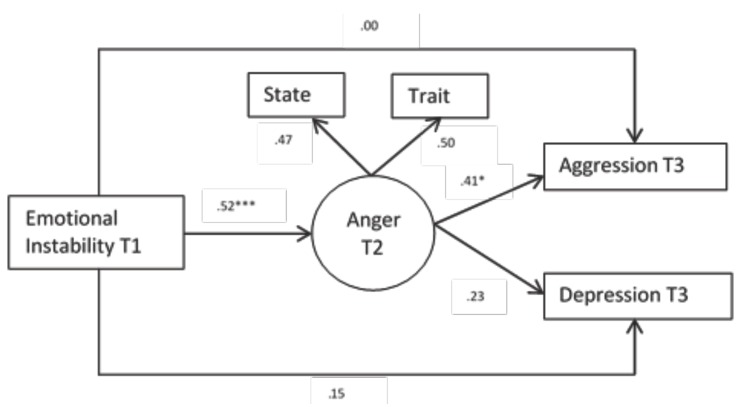
Multigroup structural equation model for boys. Standardized values. Note. ***p < .001, **p < .01, *p < .05. T1= Time 1, T2= Time 2 and T3= Time 3.

The results show, in the boys’ model, that emotional instability in interval 1 predicts in an indirect way the physical and verbal aggression in interval 3 through anger state-trait in interval 2 (see Fig. 1).

**Figure 2:**
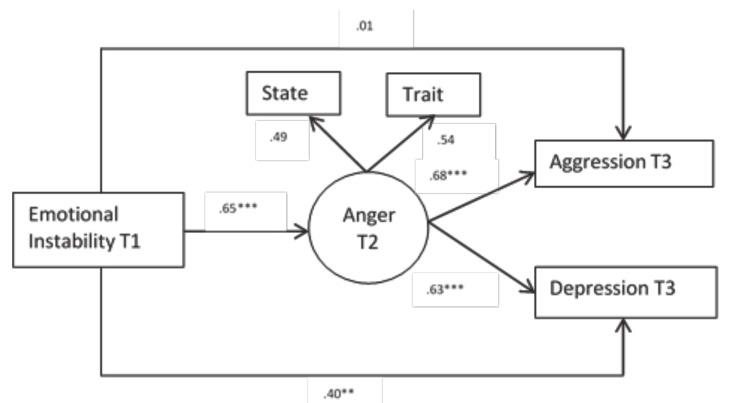
Multigroup structural equation model for girls. Standardized values. Note. ***p < .001, **p < .01, *p < .05. T1= Time 1, T2= Time 2 and T3= Time 3.

In the case of the girls, the results show that emotional instability in interval 1 predicts directly the depression in interval three. However, it is a strong predictor of anger, both state and trait in interval 2. The same way as in the boys, anger in interval 2 is also a mediator between emotional instability and physical and verbal aggression. Also, contrary to what happens with the boys, anger in interval 3 is a mediator between emotional instability and depression in interval 3 (see Fig. 2).

In turn, the girls and boys’ models differ in the predictive power because in the girls it is higher in all instances both in the direct prediction of depression as well as in it mediator function of anger (see Fig. 2).

To sum up we can defend that an internalised variable, like emotional instability, is relevant to prevent the appearance of depression directly in girls and also the later appearance of aggression as long as anger mediates, in both sexes.

## Discussion and Conclusions

The longitudinal data shows two things: First of all, we see a strong consistency of the instruments used and secondly, also, a permanency of the alterations that have been studied. This is consistent with the literature that shows that in adolescence, its related problems, as well as having a high prevalence, cannot be interpreted, in any way, as a transitory alteration (7).

As we have already seen, the correlation between aggression and depression isn’t important enough to become a powerful predictor either near in time or later on. This means that, despite the existence of a specific association, it is too weak and, in fact, in the literature it appears as a variable association (20).

Here in almost all variables the correlations are much more powerful when they coincide in time and this connection weakens as the evaluations are further in time, however, the data of this study about the relation between emotional instability and depression show that the power of this relationship it is maintained throughout time, which is a new contribution.

The correlation between anger and the rest of the variables seems much more intense with aggression than with any other. Here a much more intense correlation will be made based on the proximity in time as it is more frequent (3). The fact that the correlation between anger and depression is weaker than the correlation with aggression is something that appears in the literature repeatedly (21).

It becomes apparent that for the purpose of emotional interaction, anger trait represents a more important role than the anger state which we can find consistently in previous research about emotions in adolescence (22). This is perfectly coherent given that anger state is crucial and it is not permanent and therefore it has to vary much more easily than anger trait (22,23).

The structural equations reveals that with the mediation of anger, emotional instability acquires a higher predictive power as time goes by both in boys and girls. There is a great structural parallelism but with quantitative differences. These results can also be found in other investigations (24).

In regards to anger, there appears a great balance in the role it performs in the case of boys and girls. This implicitly means that the control of anger becomes an important goal to control the rest of the negative affects. Theoretically this would only justify the need to include anger in the negative affects model from the Clark and Watson model, as it occupies a special place in the relation with depression.

To sum up, in the models it becomes apparent that instability predicts depression and anger, but not aggression, the latter only appears if instability is associated to anger.

The limitations of this research, as it happens in every case, is the absence of some conditions that could improve the conclusions, like, for example, to have had included different sources of information, however, as mainly the emotional variables have been studied, the selection of the self-report has undeniable advantages.

From all that has been presented we can extract several conclusions: First of all and, as it is obvious, there is an important difference between boys and girls in the emotional field. However, these differences are more quantitative than qualitative.

On the other hand we can state that the prediction of emotions of the internalised kind can predict those internalised directly, but also the indirectly internalised if we take anger into account. It is obvious that all this has consequences in the preparation of all programmes that try to establish an emotional control on adolescents, as not only has to be taken into account as a direct goal the control of externalised emotions like anger, but internalised emotions like emotional instability have to be taken into account also.

Furthermore, it is also made apparent that not only the punctual explosions and externalisation of anger have to be worked on, but the temperamental aspects which are the base of anger trait have to be worked on too. This is in line with a more globalized concept of control of emotions to make it effective in the long term.
